# Health-Related Quality of Life in Pregnant Women With Gestational Hypertension: A Systematic Review

**DOI:** 10.7759/cureus.61340

**Published:** 2024-05-30

**Authors:** Mohamad Zakour Khadari, Hadzliana Zainal, Nur Aizati Athirah Daud, Abubakar Sha'aban

**Affiliations:** 1 Clinical Pharmacy, Universiti Sains Malaysia, George Town, MYS; 2 Population Medicine, Cardiff University, Cardiff, GBR

**Keywords:** complicated pregnancy, gestational hypertension, quality of life, pregnancy-induced hypertension, pregnancy, health-related quality of life

## Abstract

This review seeks to evaluate the levels of health-related quality of life (HRQoL) among pregnant women experiencing pregnancy-induced hypertension (PIH). It also aims to identify the specific aspects of HRQoL most impacted by PIH during pregnancy and determine the existence of effective interventions to enhance the HRQoL of these pregnant women. A systematic literature search was conducted in the following databases: PUBMED, SCOPUS, Google Scholar, and EMBASE using the following keywords: Health-related quality of life; pregnancy; pregnancy-induced hypertension; quality of life; gestational hypertension. Among the 32 studies assessed, only eight met the criteria for inclusion, exhibiting a good quality based on assessment with both AXIS (Appraisal Tool for Cross-Sectional Studies) and CASP (Critical Appraisal Skills Programme) checklists. The findings indicate a decline in HRQoL among pregnant women with gestational hypertension, notably affecting both physical and mental dimensions. Furthermore, some studies provided recommendations for interventions that healthcare professionals could employ to improve poor HRQoL levels. Limited research has focused on the HRQoL in pregnant women with PIH. Compared to their healthy counterparts, pregnant women experiencing PIH exhibit a decrease in their HRQoL. It's crucial for healthcare practitioners to proactively address the HRQoL of these pregnant women using effective strategies to mitigate this decline. This approach aims to safeguard both pregnant women and their fetuses from potential complications associated with lower HRQoL levels.

## Introduction and background

Hypertensive disorders in pregnancy may include gestational hypertension, which is usually diagnosed after the 20th gestational week; chronic hypertension, which is diagnosed before conception or before the 20th gestational week; and preeclampsia, which appears after the 20th gestational week and is associated with proteinuria [1,2].

About 10% to 22% of pregnant women are prone to any type of hypertensive disorder during pregnancy. These statistics are expected to increase tremendously as a result of a rise in the prevalence of chronic hypertension risk factors, including obesity and metabolic syndrome but not including increasing maternal age [3]. As a result, the number of pregnant women with chronic hypertension is likely to expand globally, which not only represents a difficulty for the pregnant mother but may lead to serious consequences. It may also threaten the pregnant woman’s life, because gestational hypertension is considered one of the leading causes of death among pregnant women in developing countries [2,4]. Moreover, this condition is associated with long-term complications that include heart diseases such as chronic hypertension, in addition to some other negative effects on the fetus. Other fetal complications include low birth weight, intrauterine growth retardation, or even death of the newborn [4].

In recent years, health-related quality of life (HRQoL) has become one of the most frequently used metrics to assess a patient’s health status and the severity of their ailment. Individuals’ HRQoL varies depending on their demographics as well as other aspects such as their social and economic situations, level of education, and level of health literacy [5].

Quality of life, according to the World Health Organization, encompasses people’s opinions of their position in life in terms of their culture, value system, goals, expectations, standards, and priorities [5]. At this time, corresponding to current scientific concepts, assessing and measuring the quality of life gives critical information about the health status of individuals, as well as about how it can be used to improve their health if it is raised to decent levels. Accordingly, measuring the quality of life during pregnancy is critical for using strategies for the prevention and treatment of some pregnancy-related disorders [6].

The subjective appraisal of one’s present health status, as well as health care and health promotion activities, is the focus of HRQoL, which has become a valuable tool for assessing a person’s current health status. In medical and public health research, it has been established as an outcome variable and health status indicator [7].

Pregnancy alone constitutes an obstacle for a pregnant woman that limits her ability to carry out her daily activities normally, not to mention the psychological and mental burden it poses. Thus, the presence of a chronic illness during pregnancy makes women more vulnerable to lower levels of HRQoL, which can interfere with the outcome of pregnancy for both the woman and her fetus [8,9]. As an example of how low levels of HRQoL can affect the outcome of gestation, a poor quality of life has been associated with cases of preterm labor and intrauterine growth restriction for pregnant women [10].

Despite the significance HRQoL has to the health of individuals in general and in vulnerable groups of patients such as pregnant women in particular, there has been relatively little investigation into the issue of HRQoL in pregnant women experiencing one of the forms of gestational hypertension.

Considering the importance of HRQoL as an indicator of people’s health status and taking into account the prevalence of hypertension in pregnancy and its serious health consequences for the pregnant woman and her fetus, this review aims to shed light on studies that have handled the topic of HRQoL in pregnant women with gestational hypertension. The results of the review aim to encourage further investigations and initiatives addressing this topic.

## Review

Methodology

Data Sources

The following search engines were used to conduct systematic searches: PubMed, Embase, Google Scholar, and Scopus (Table 1). The MESH database was used to construct the terminology employed in the research process to attain the highest number of studies that simulated the area of interest. Each of the databases used in the systematic search adopted these terms. Because the concept of HRQoL is not commonly addressed throughout pregnancy, the studies included were not constrained to a set time range. This review only includes articles published in the English language.

**Table 1:** Search Strategy

Database	Keywords
PubMed	(((Hypertension(s), Pregnancy Induced) OR (Pregnancy-Induced Hypertension)) OR (Gestational Hypertension)) AND (((Quality of Life) OR (Health-Related Quality of Life)) OR (HRQOL)).
SCOPUS	TITLE-ABS-KEY ( ( hypertension, AND pregnancy AND induced OR pregnancy-induced AND hypertension OR gestational AND hypertension) AND ( quality AND of AND life OR health-related AND quality AND of AND life OR hrqol ) )
Google Scholar	With all the words: Pregnancy Induced Hypertension AND Quality of Life with at least one of the words: “Pregnancy Induced Hypertension” “Quality of Life”
EMBASE	(((pregnancy.sh OR pregnancy.ab,ti OR pregnancy-induced.ab,ti OR “gestational-pregnancy”ab,ti,tw)) AND ((hypertension.ab,ti. OR hypertension.sh.)). AND ((“quality of life”.ab,ti,tw. OR “quality of life”.sh. OR “health-related quality of life”.ab,ti,tw.)))

Four authors independently reviewed the abstracts of the studies featured in the search. Studies matching the inclusion criteria were reviewed for full text.

Inclusion and Exclusion Criteria

The inclusion criteria consist of pregnant women at any stage of pregnancy, articles published in the English language, various study designs, HRQoL or quality of life as an outcome, and pregnant women with any type of gestational hypertension. The criteria also include assessments of the effect of gestational hypertension on HRQoL, the effect of HRQoL on developing any form of gestational hypertension, and the role of HRQoL in the treatment of gestational hypertension.

The exclusion criteria involve measuring HRQoL in healthy pregnant women and measuring HRQoL in women with multiple pregnancy disorders, such as gestational diabetes and gestational hypertension.

Data Extraction

The relevant materials for the research topic, such as the characteristics of the study and quantitative results, in addition to other data that were inventoried, such as the country in which the study was conducted and the year of the study, were extracted based on a predetermined strategy by the author.

Quantitative data were retrieved in the same manner as provided in the study, including HRQoL levels for pregnant women suffering from one type of hypertension disorder, pregnancy outcomes, and the health condition of the fetus at delivery. The data were not quantitatively summarized, given the diversity of the characteristics of the studies and the methods for representing data in the original papers.

Quality Assessment

To assess the quality of the studies included in the review, standardized checklists were used to certify the papers’ quality. The AXIS (Appraisal Tool for Cross-Sectional Studies) checklist is a standardized tool designed to check the quality of cross-sectional studies and the risk of bias. It includes 20 questions that cover various aspects of cross-sectional studies; “Yes/No” or “Don’t know/Comment” are used to answer each question. AXIS does not give a comprehensive evaluation of the research [11].

For studies that adopted a cohort design, the CASP (Critical Appraisal Skills Programme) checklist was used to evaluate the quality of the research. This evaluation tool contains 12 questions that focus on the study’s outcomes, and if the results are valid and can be used broadly, the first two questions are screening questions that may be rapidly answered. If the answer to both is “yes,” it is worth proceeding with the remaining questions [12]. Four independent reviewers graded the study’s quality (MZK, HH, AS, and NAD).

Results

Study Selection

The authors examined the abstracts of the scanned studies to determine which papers of interest should be included in the review. The included articles were selected based on the previously determined inclusion criteria; topics addressing the quality of life in hypertensive pregnant women were considered relevant to the review context, and other topics discussing a different aspect of the health of pregnant women were excluded.

**Figure 1:**
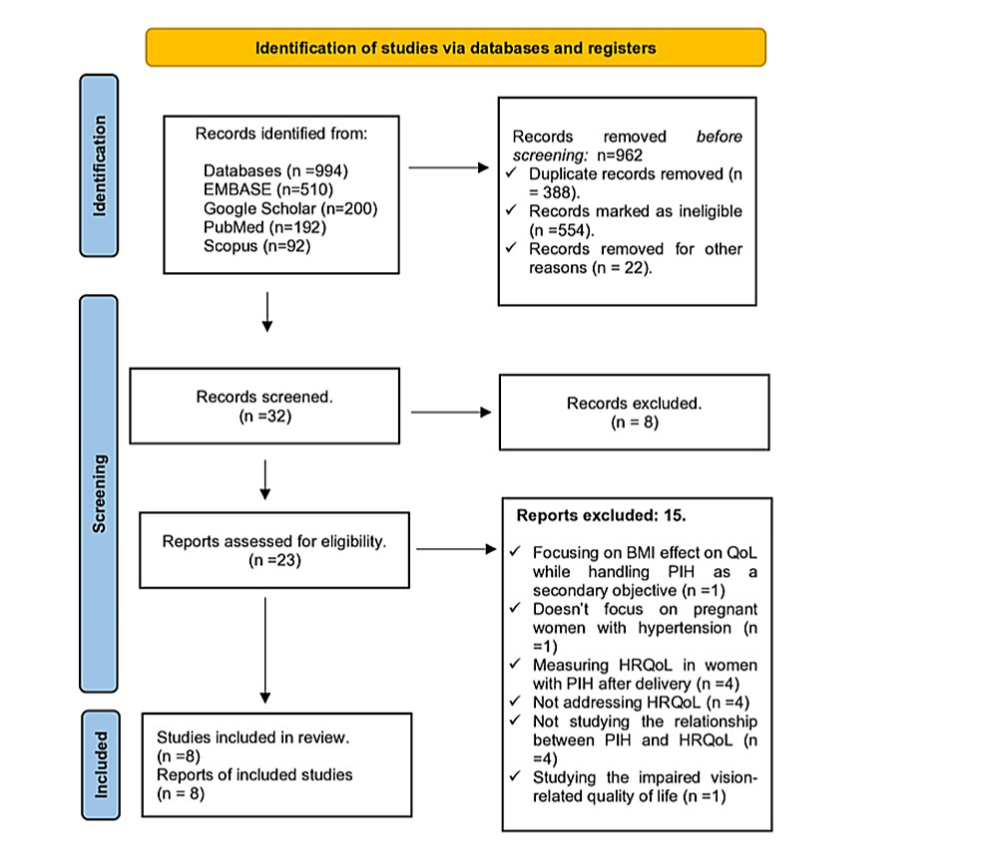
Review Flowchart BMI: body mass index; QoL: quality of life; HRQoL: health-related quality of life; PIH: pregnancy-induced hypertension

Figure 1 illustrates the flowchart of the study selection procedure. A total of 994 papers were identified during the primary search process and distributed as follows: EMBASE (n = 510), Google Scholar (n = 200), PubMed (n = 192), and Scopus (n = 92). Of the total, 962 studies did not match the inclusion criteria, leaving 32 for full-text review. Out of these 32 articles, eight were removed because of duplication, 15 were eliminated for the reasons mentioned in Figure 1, and ultimately eight studies were selected to be included in this systematic review.

Study Characteristics

The characteristics of the studies included in the systematic review are summarized in Table 2. The study formats varied between a cross-sectional design (three of the studies) [13-15], a cohort design (three of the studies) [16-18], one longitudinal prospective study on women with complicated pregnancies [19], and one observational study (a web survey) [20].

**Table 2:** Study Characteristics

First Author	Year	Countries	Study Design	Eligibility Criteria	Gestational Hypertension/Preeclampsia Definition	Study Outcome	N in Analysis	Sampling and Recruitment
Machado et al. [13]	2020	Brazil	Cross-sectional	Hypertensive pregnant women with uncontrolled blood pressure	Not Mentioned	Quality of life of hospitalized pregnant women with preeclampsia in comparison to that of healthy pregnant women	58	Convenience sampling in hospital
Medeiros Falcão et al. [14]	2016	Brazil	Cross-sectional	≥18 years with established hypertension without comorbidities (diabetes, asthma, acquired or congenital heart disease)	Gestational hypertension: blood pressure ≥ 140/90 mmHg without proteinuria, diagnosed for the first time during pregnancy, after the 20th gestational week	Association between hypertension in pregnancy and women’s quality of life (QoL) and the variation in the quality of life	389	Convenience Sampling
Chmaj-Wierzchowska et al. [15]	2021	Poland	Cross-sectional	Women with pathological pregnancies who had pregnancy-induced hypertension, fatal hypotrophy, cholestasis, or gestational diabetes mellitus, in addition to healthy pregnant women for the control group	Not mentioned	The incidence of health problems in pregnant mothers and the influence of these problems on their daily functioning	232	Convenience sampling in hospital
Hoedjes et al. [16]	2011	Netherlands	Multicentre cohort study	Women who had given birth between February 2007 and June 2009, if their pregnancy had been complicated by preeclampsia, intrauterine growth restriction, or gestational diabetes	Preeclampsia: blood pressure of more than or equal to 140⁄ 90 mmHg in combination with proteinuria (defined as ±300 mg⁄ day of urinary protein loss) after 20 weeks of gestation	1) Changes in all domains of health-related quality of life between 6 and 12 weeks postpartum after mild and severe preeclampsia; 2) The extent to which health-related quality of life differed after mild and severe preeclampsia; and 3) Factors contributing to such differences in health-related quality of life	174	Convenience Sampling in hospital
Pan et al. [17]	2021	China	Cohort study	Patients diagnosed with gestational hypertension	Gestational hypertension: (systolic/diastolic blood pressure ≥140/90 mmHg) after ≥20 + 0 weeks gestation	The efficacy of comprehensive care during the nursing process of a parturient with gestational hypertension and postpartum depression	70	Convenience sampling in hospital
Stern et al. [19]	2013	Austria	Cohort study	Women with gestational hypertension	Not Mentioned	The physical and mental health-related quality of life (HR-QoL) in women after PE and the impact of contributing factors	95	Convenience sampling in the clinic
Postma et al. [20]	2013	USA	Observational study survey	Women with pregnancy-related hypertensive disorder, including preeclampsia (toxemia), HELLP syndrome, or pregnancy-induced hypertension (PIH) during any of their pregnancies	Not mentioned	Scope of perceived neurocognitive and psychosocial problems as well as quality-of-life following preeclampsia	1308	Web-based survey
Mautner et al. [21]	2009	Austria	A prospective, longitudinal study	Women with complicated pregnancies between 24 and 37 weeks of gestation, sufficient German language skills, and women who planned to deliver at the gynecology clinic (where the study was conducted)	Gestational hypertension: blood pressure ≥ 140/90 mmHg without proteinuria, diagnosed for the first time during pregnancy, after the 20th gestational week	The influence of hypertensive disorders, gestational diabetes, and preterm birth as risk factors for health-related quality of life (HRQL) and depressive symptoms	90	Convenience sampling in gynecology clinic

The studies included in the review were conducted in various locations, such as several European countries, South America, North America, and Asia. The sample sizes ranged from 58 to 1308 pregnant women with gestational hypertension.

The inclusion criteria of the articles differed from one another. Still, one criterion common to all studies is that the women included in the research must have been diagnosed with gestational hypertension or one of its forms. However, one of these studies included women who had completed their pregnancies and had experienced gestational hypertension [16]. Some of these studies required the women to have more than one pregnancy complication (gestational hypertension) because these studies required the female participants to have gestational diabetes as well. Further details on the included articles are available in Table 2.

In the reviewed studies, various measures were employed to assess the quality of life in pregnant women; however, almost every single study used a different type of questionnaire to measure the quality of life.

Two studies used the WHO Quality of Life questionnaire (WHOQOL-BREF) [13,22]. The short-form health survey known as SF-HLS was implemented in two of the studies, once in its full version translated into German, RAND SF-36, [16] and in another paper in its shortened version, SF-12 [18].

In addition to the use of numerous other instruments such as the Index of Quality of Life for Various Populations (IQVFP), Nottingham Health Profile (NHP), General Quality of Life Inventory-74 (GQOLI-74), and Cognitive Failures Questionnaire (CFQ), all these tools were designed to assess the quality of life in individuals and all serve the same function. Still, the instruments vary in the number of questions and elements they examine. Table 3 lists the tools used in the papers under consideration and briefly explains each.

**Table 3:** Tools Used in the Studies IQVFP: Index of Quality of Life for Various Populations; NHP: Nottingham Health Profile; RAND 36-item SF-36: Short Form Health Survey; GQOLI-74: General Quality of Life Inventory-74; SF-12: Short Form-12 Health Survey; CFQ: Cognitive Failures Questionnaire; WHOQOL-Bref: World Health Organization Quality of Life-BREF; QoL: quality of life; HRQoL: health-related quality of life

Tools	Descriptions	Scoring
IQVFP [14]	This tool evaluates QoL in both healthy persons and those with an illness and contains two components: the first assesses satisfaction in various dimensions, and the second analyses the relevance of each of these domains for the responder.	The scores range from zero to 30, and the higher values indicate a higher QoL.
NHP [15]	This was generated to assist patients in making a broad assessment of their subjective health state in a variety of areas.	With a total score of 100, higher scores link to greater levels of health.
RAND 36-item SF-36 [16]	This survey measures the quality of life and uses additional summary measures: a physical component scale (which includes the subscales of physical functioning, physical role, bodily pain, and general health) and a mental component scale (consisting of vitality, social functioning, role emotional, and mental health).	Scales are transformed to ranges of 0 to 100; higher scores indicate a better health-related quality of life.
GQOLI-74 [17]	GQOLI-74 is a generalized QoL questionnaire with 20 components, each reflecting a different facet of QoL into four dimensions: physical function, psychological function, social function, and material well-being.	Given a total of 100 points, a higher score indicates a better quality of life.
SF-12 [18]	This examines the physical and mental HRQoL scores extracted from the SF-36 questionnaire. These health status tools are also used to assess the impact of various disorders on the HRQoL of patients.	Scores range between 0 and 100; higher scores indicate a better health-related quality of life.
CFQ [20]	This assesses how often over the past six months errors were committed in daily tasks of everyday life; it has 25 questions that are graded on a five-point scale.	Given a total score of 100, higher scores on the CFQ indicate more cognitive failures.
WHOQOL-Bref (Machado et al., 2020) [22]	This tool assesses the general quality of life of patients through four domains: general quality of life, the physical domain, the psychological domain, and the domain of personal relationships.	Of a total score of 100, quality of life scores are categorized as follows: 0-20, very poor; 21-40, poor; 41-60, neither bad nor good; 61-80, good; and 81-100, very good.

HRQoL in Pregnant Women With Gestational Hypertension

The results of the studies included in the review varied depending on the study’s objectives, but there was some agreement that pregnant women with gestational hypertension had a lower quality of life compared to their healthy pregnant counterparts. However, one study from a long series of trials discovered no difference in the quality of life between pregnant women with and without gestational hypertension, such that pregnant women in both groups (healthy pregnancy and hypertensive) scored adequately in all domains of the WHOQOL-BREF questionnaire [13].

In contrast, even though the same tool was implemented by Postma et al., their observational study of 2013 revealed that pregnant women who had previously suffered from preeclampsia suffered from a degenerated quality of life and scored significantly lower in comparison to those with normotensive pregnancies [20].

These results are in line with studies that employed the SF-HLS in its two different formats (SF-36 and SF-12) [16,18]. Both studies reported low levels of HRQoL in pregnant women who were suffering from preeclampsia, and this reduction in HRQoL levels was observed in all aspects of quality of life.

Medeiros Falcão et al. reported findings similar to those of previously mentioned articles, using the IQVFP tool. They concluded that high blood pressure during pregnancy is responsible for psychological and physical variations in pregnant women. This alteration in the physiology and psychology of pregnant women can cause a decline in their quality-of-life levels; the lowest score was recorded in the health and function aspect at 17.63 and the highest score of 26.0 was recorded in the family domain of the IQVEP instrument [14].

Healthcare Providers’ Role in Improving HRQoL of Women With Gestational Hypertension

The reviews included studies that looked at more than just the quality of life of pregnant women with gestational hypertension. Some of the studies looked at the role of healthcare providers and the medical care given to these women, along with how the care affected their quality of life and how it helped them have healthier pregnancies.

Individually tailored medical care can spare pregnant women the consequences of hypertension disorder while pregnant; they can end up having normal pregnancy experiences just like healthy women. This was reported in a study conducted by Machado et. al., who compared the quality of life of pregnant women with and without gestational hypertension; the results showed no difference in the total quality of life between the two study arms. The author ascribed these findings to the hospital’s involvement in providing health care to the gestational hypertension group [13].

Several studies concluded that blood pressure disorders during gestation directly account for the deterioration in the HRQoL of pregnant women [23,24]. Hoedjes et al. urged health professionals to raise the levels of psychological care provided during pregnancy and to continue after delivery, especially for those women with pregnancy-related complications [16].

Moreover, Chmaj-Wierzchowska et al. emphasized the role of medical care provided by healthcare specialists and used the NHP as a tool to assess changes in the psychological functioning of pregnant women with complications. They highlighted the need to personalize the health care provided to hypertensive pregnant women based on the requirements of each patient, given the importance of this matter in raising the quality of life in this group of pregnant women [15].

Cognitive Functions and HRQoL in Pregnant Women With Gestational Hypertension

Among the different aspects of HRQoL, the reviewed studies focused on the psychological factors more than the others. In an investigation into the impact of severe preeclampsia on quality of life, it was noticed that, in addition to the effects on mental health, preeclampsia had an impact on individuals’ behavior and social relationships, because these individuals showed high levels of depression and nervousness, which negatively impacted their daily activities and ability to perform their jobs [16].

The findings of a study by Pan et al. were similar, in that high levels of depression were present in pregnant women with gestational hypertension, and these levels of depression were associated with aggressive behaviors that could lead to self-harm, as well as to slowing the psychological and physical recovery process for this group of pregnant women [17].

Additionally, the results of a study aimed at evaluating the mental states of pregnant women with hypertension disorders showed that the state of depression may persist for a long time after labor and delivery. The depression lasted for years in some cases and interfered with the mental states of the women and affected their relationships with their infants. In addition, these altered mental states were responsible for prolonging the degeneration of the women’s quality of life even after delivery [20].

Multiple pregnancies were also shown to affect the quality of life among women with gestational hypertension. One study showed that the levels of mental and cognitive health are worse in women who have given birth more than once compared to women who have not given birth or have given birth once at most. The reason is that women with multiple pregnancies are responsible for several children, which lowers the quality of life and cognitive functions, a result exacerbated by the presence of preeclampsia [18].

Another factor that interferes with cognitive functions in pregnant women with gestational hypertension is the body mass index (BMI). Elevated values of the BMI are negatively reflected in the mental states of pregnant women. According to the results of the study conducted by Chmaj-Wierzchowska and colleagues, higher levels of BMI were associated with worse values of HRQoL, particularly the aspects of psychological functioning, physical activity, and social isolation. These effects were shown to be milder in healthy pregnancies than in pathological pregnancies such as those affected by gestational hypertension or gestational diabetes [15].

Quality Assessment

All studies that used a cross-sectional design received a total score of 13 to 16 points out of a possible 20 on the AXIS checklist. In studies that used a cohort design, the tool used was CASP. This tool does not use a points system but highlights a group of factors divided into sections; any of these factors can be critical in a cohort design. The essential points listed in the CASP evaluation tool were all covered in the cohort studies included in this study.

Almost all the papers included in the study met the quality criteria established specifically for this systematic review. These requirements include identifying the study’s target group explicitly and in-depth as well as employing dependable tools to collect data from the investigated sample. Another parameter reported in all included studies is the use of a precision estimate (e.g., p-values) to define the significance levels mentioned in the methods section or in the results. It is crucial to disclose limitations and explanations of limitations in any research project, and they have been explicitly described in all the studies covered except for two.

The studies did not meet some quality requirements; for example, only two studies out of eight presented the requisite grounds to justify the sample size used, and in four out of eight, convenience sampling was the chosen sampling technique.

Overall, the studies included in the review were of moderate to good quality based on the criteria and tools used to make that assessment.

Discussions

This systematic review is the first to address the issue of HRQoL in pregnant women with one of the types of gestational hypertension. Eight studies were identified as relevant to our review, and these papers covered several different aspects of the reviewed topic, such as distinct forms of gestational hypertension, the components of quality of life in pregnant women most impacted by gestational hypertension, and the necessity of guided medical treatment for this group of pregnant women.

As for the first objective of this review, most of the studies obtained the same results regarding the effect of PIH on HRQoL: that gestational hypertension impairs the quality of life in pregnant women, and this impairment may last even beyond the delivery in some cases [22,25,26]. However, one paper out of the eight found no difference in the quality of life between healthy pregnant women and those suffering from gestational hypertension. The authors of the study explained the lack of difference in the quality of life by the fact that the pregnant women with gestational hypertension were under direct care in the hospital, probably leading to an improvement in their mental health as well as in their quality of life, which rose to the same levels as in healthy pregnant women [27].

When the HRQoL is degenerated by gestational hypertension, the physical, psychological, and social components are most affected. The physical aspect is one of the areas of health that is prone to alterations throughout normal pregnancy, but it deteriorates significantly in the presence of gestational hypertension [28]. To improve this aspect of HRQoL, researchers have investigated the role of exercise for pregnant women and have concluded that simple aerobic exercises can improve the physical condition of pregnant women and help prevent preeclampsia [29].

One repeated finding across the reviewed studies is that the decline in quality of life for pregnant women with gestational hypertension is linked to the onset of chronic depression, and this depression in some cases will last beyond the pregnancy [30]. The occurrence of depression in pregnant women suffering from pregnancy-related disorders is common, with an incidence ranging from 6% to 54.5%. In addition to slowing the recovery process and returning the quality of life to normal, depression carries negative health consequences for the mother, including decreased milk production, fetal growth restriction, psychological effects, and aggressive behavioral changes which can include causing injury to self or others [20,17].

Targeted health care has been studied for its role in promoting HRQoL and in reducing complications of gestational hypertension (such as preeclampsia and fetal complications). Regular group counselling sessions or regular meetings with a psychologist (for women with gestational hypertension) will increase the levels of HRQoL because of the psychological comfort created by the fact that the pregnant woman’s health state is monitored by experts in their area, avoiding negative health effects [18].

Indicating the importance of the health care provided to pregnant women who suffer from gestational hypertension, the results of a study comparing healthy pregnant women with their peers who suffer from gestational hypertension found no difference in the quality of life in both arms of the study, and the authors have concluded that this is because pregnant women with hypertensive disorders are subject to health monitoring and direct medical supervision by specialists [13].

Medical follow-ups can take several forms and can be continued during the postpartum period, so some studies have recommended the role of mental health follow-ups and postpartum psychotherapy sessions for those women who have had a near-death experience caused by pregnancy or for women who had their fetuses admitted to intensive care units as a result of preeclampsia [16].

In addition, psychological care has other benefits during pregnancy, such as boosting thyroid function and reducing postpartum eclampsia; these therapies have been shown to improve the mental state of pregnant women following childbirth, as well as their HRQoL [18,17].

Literature have identified the factors that decrease the quality of life for pregnant women diagnosed with gestational hypertension. One of these factors is psychological. If a pregnant woman becomes aware that she has high blood pressure and that her pregnancy has been classified as risky, her concern could deteriorate her quality of life, which could already be low due to physiological and psychological changes carried by pregnancy [31]. Her anxiety and depression levels may also increase once she considers her newly diagnosed health status and its potential consequences [13].

Moreover, among the possible factors responsible for reducing HRQoL in pregnant women with gestational hypertension are the admission of neonates to intensive care, neonatal mortality, caesarean section, and preterm birth, which contribute to the decline in quality of life in the postpartum period [16].

Another factor that may influence the HRQoL of pregnant women is sleep quality. Poor sleep levels and poor sleep quality have been connected to depression as well as to the poor quality of life in pregnant women, according to a study conducted on pregnant women diagnosed with gestational hypertension and admitted to the hospital for monitoring [17].

Postma et al. identified another possible factor responsible for the degeneration of the HRQoL of pregnant women with gestational hypertension. This factor is the impaired cognitive function and the decline in social performance as a result of having preeclampsia. Impaired cognitive function includes difficulties in concentrating and mental depression, which can contribute to a worsening of HRQoL in this group of pregnant women [20].

Limitations

Even completed works have limitations or flaws, and identifying these limits is necessary if other researchers are to be guided to avoid these constraints.

First, the quality of the included studies was average, particularly those with a cross-sectional design, which had a total score of 13-16 out of 20 on the AXIS checklist. As for the research with a cohort design, although the assessment tool did not include a specific points system, all the papers that adopted this design met all the requirements mentioned in the CASP checklist.

Second, the definitions of quality of life fluctuated among the studies, such that some studies measured the quality of life in general, whereas others measured HRQoL. Both definitions replicate an individual’s health characteristics, but quality of life is a broader term. The concept of HRQoL is more health-related, but that does not imply that the concept of quality of life is less health-related. Quality of life can be utilized in research to evaluate individual health.

Last, some researchers investigated the quality of life of pregnant women with gestational hypertension during pregnancy, but other researchers examined the quality of life during the postpartum period. This variety in these studies helps provide a full picture of life quality across a longer period.

## Conclusions

Gestational hypertension has a detrimental impact on the quality of life of pregnant women. This effect manifests itself in aspects of mental, social, and physical quality of life; in addition, psychological conditions such as depression may be associated with gestational hypertension, thus creating another burden for pregnant women and for health-care providers.

The role and relevance of a patient-designed medical treatment are highly appreciated in enhancing the quality of life and consequently the outcomes of delivery. The significance of evaluating the quality of life in pregnant women with gestational hypertension stems from the possible health concerns that could be averted if the quality of life is assessed and all required medical precautions are adopted. These risks are then linked to the health of pregnant women and their pregnancy outcomes. Finally, further research and clinical investigations are needed to identify the type and duration of suitable medical treatments for this patient group.

## Appendices

**Table 4:** PRISMA Checklist PRISMA: Preferred Reporting Items for Systematic Reviews and Meta-Analyses

Section and Topic	Item #	Checklist item	Location where item is reported
TITLE			Cover page
Title	1	Identify the report as a systematic review.	
ABSTRACT			
Abstract	2	See the PRISMA 2020 for Abstracts checklist.	2
INTRODUCTION			
Rationale	3	Describe the rationale for the review in the context of existing knowledge.	4
Objectives	4	Provide an explicit statement of the objective(s) or question(s) the review addresses.	4
METHODS			
Eligibility criteria	5	Specify the inclusion and exclusion criteria for the review and how studies were grouped for the syntheses.	6
Information sources	6	Specify all databases, registers, websites, organisations, reference lists and other sources searched or consulted to identify studies. Specify the date when each source was last searched or consulted.	5
Search strategy	7	Present the full search strategies for all databases, registers and websites, including any filters and limits used.	7
Selection process	8	Specify the methods used to decide whether a study met the inclusion criteria of the review, including how many reviewers screened each record and each report retrieved, whether they worked independently, and if applicable, details of automation tools used in the process.	8
Data collection process	9	Specify the methods used to collect data from reports, including how many reviewers collected data from each report, whether they worked independently, any processes for obtaining or confirming data from study investigators, and if applicable, details of automation tools used in the process.	9
Data items	10a	List and define all outcomes for which data were sought. Specify whether all results that were compatible with each outcome domain in each study were sought (e.g. for all measures, time points, analyses), and if not, the methods used to decide which results to collect.	10
	10b	List and define all other variables for which data were sought (e.g. participant and intervention characteristics, funding sources). Describe any assumptions made about any missing or unclear information.	8
Study risk of bias assessment	11	Specify the methods used to assess risk of bias in the included studies, including details of the tool(s) used, how many reviewers assessed each study and whether they worked independently, and if applicable, details of automation tools used in the process.	8
Effect measures	12	Specify for each outcome the effect measure(s) (e.g. risk ratio, mean difference) used in the synthesis or presentation of results.	12
Synthesis methods	13a	Describe the processes used to decide which studies were eligible for each synthesis (e.g. tabulating the study intervention characteristics and comparing against the planned groups for each synthesis (item #5)).	12
	13b	Describe any methods required to prepare the data for presentation or synthesis, such as handling of missing summary statistics, or data conversions.	12
	13c	Describe any methods used to tabulate or visually display results of individual studies and syntheses.	12
	13d	Describe any methods used to synthesize results and provide a rationale for the choice(s). If meta-analysis was performed, describe the model(s), method(s) to identify the presence and extent of statistical heterogeneity, and software package(s) used.	No meta analysis was performed
	13e	Describe any methods used to explore possible causes of heterogeneity among study results (e.g. subgroup analysis, meta-regression).	
	13f	Describe any sensitivity analyses conducted to assess robustness of the synthesized results.	
Reporting bias assessment	14	Describe any methods used to assess risk of bias due to missing results in a synthesis (arising from reporting biases).	
Certainty assessment	15	Describe any methods used to assess certainty (or confidence) in the body of evidence for an outcome.	
RESULTS			
Study selection	16a	Describe the results of the search and selection process, from the number of records identified in the search to the number of studies included in the review, ideally using a flow diagram.	11
	16b	Cite studies that might appear to meet the inclusion criteria, but which were excluded, and explain why they were excluded.	11
Study characteristics	17	Cite each included study and present its characteristics.	13-21
Risk of bias in studies	18	Present assessments of risk of bias for each included study.	
Results of individual studies	19	For all outcomes, present, for each study: (a) summary statistics for each group (where appropriate) and (b) an effect estimate and its precision (e.g. confidence/credible interval), ideally using structured tables or plots.	13-21
Results of syntheses	20a	For each synthesis, briefly summarise the characteristics and risk of bias among contributing studies.	
	20b	Present results of all statistical syntheses conducted. If meta-analysis was done, present for each the summary estimate and its precision (e.g. confidence/credible interval) and measures of statistical heterogeneity. If comparing groups, describe the direction of the effect.	No meta analysis was performed
	20c	Present results of all investigations of possible causes of heterogeneity among study results.	
	20d	Present results of all sensitivity analyses conducted to assess the robustness of the synthesized results.	
Reporting biases	21	Present assessments of risk of bias due to missing results (arising from reporting biases) for each synthesis assessed.	
Certainty of evidence	22	Present assessments of certainty (or confidence) in the body of evidence for each outcome assessed.	
DISCUSSION			
Discussion	23a	Provide a general interpretation of the results in the context of other evidence.	30
	23b	Discuss any limitations of the evidence included in the review.	33
	23c	Discuss any limitations of the review processes used.	33
	23d	Discuss implications of the results for practice, policy, and future research.	34
OTHER INFORMATION			
Registration and protocol	24a	Provide registration information for the review, including register name and registration number, or state that the review was not registered.	Review wasn’t registered
	24b	Indicate where the review protocol can be accessed, or state that a protocol was not prepared.	Review was prepared using PRSIMA guidelines
	24c	Describe and explain any amendments to information provided at registration or in the protocol.	
Support	25	Describe sources of financial or non-financial support for the review, and the role of the funders or sponsors in the review.	No financial support was provided
Competing interests	26	Declare any competing interests of review authors.	Authors declare no conflict on interest.
Availability of data, code and other materials	27	Report which of the following are publicly available and where they can be found: template data collection forms; data extracted from included studies; data used for all analyses; analytic code; any other materials used in the review.	
